# Survival, response rates, and post-transplant outcomes in patients with Acute Myeloid Leukemia aged 60-75 treated with high intensity chemotherapy vs. lower intensity targeted therapy

**DOI:** 10.3389/fonc.2022.1017194

**Published:** 2022-10-03

**Authors:** Kieran Sahasrabudhe, Ying Huang, Melanie Rebechi, Patrick Elder, Alice Mims, Sarah Wall

**Affiliations:** ^1^Division of Hematology, Department of Internal Medicine, Comprehensive Cancer Center, The Ohio State University, Columbus, OH, United States; ^2^Division of General Internal Medicine, Department of Internal Medicine, The Ohio State University, Columbus, OH, United States

**Keywords:** Acute Myeloid Leukemia, targeted therapy, intensive chemotherapy, hematopoietic stem cell transplantation, older adult oncology

## Abstract

The United States Food and Drug Administration has approved several oral, targeted therapies for the treatment of Acute Myeloid Leukemia (AML) in recent years. These agents are approved in patients with relapsed/refractory disease or as frontline therapy in patients who are ineligible for intensive chemotherapy based on age, performance status, or comorbidities. They are also being increasingly utilized frontline in patients of all ages and fitness levels through clinical trials and off label prescribing, but comparative treatment outcomes associated with intensive versus targeted therapy have not been extensively studied. We conducted a single center, retrospective analysis to address the impact of treatment intensity on survival in patients with AML aged 60-75 at diagnosis. This study included 127 patients, 73 of whom received high intensity chemotherapy at any point during treatment (any HiC) and 54 of whom received only low intensity targeted therapy (LITT only). Overall survival (OS) from treatment initiation did not differ significantly between the any HiC and LITT only groups (hazard ratio (HR) for death, 0.67; 95% CI, 0.41 to 1.09; P=0.11). The only three variables that were independently associated with superior OS were lower European Leukemia Net (ELN) risk classification, *TP53* unmutated status, and receipt of transplant. Our data suggest that baseline genomic features and receipt of transplant are more important than treatment intensity in predicting survival in this patient population. They also highlight the vital role of transplant in older patients with AML regardless of treatment intensity utilized for remission induction. Larger studies are needed to further address this question, including prospective randomized trials.

## Introduction

The United States Food and Drug Administration (FDA) has approved several oral, targeted therapies for the treatment of Acute Myeloid Leukemia (AML) in recent years. These agents are approved as frontline therapy for patients ineligible for intensive chemotherapy or as salvage therapy for patients with relapsed/refractory (R/R) disease. Some of the most commonly utilized targeted agents for AML include gilteritinib, ivosidenib, enasidenib, and venetoclax. Gilteritinib is a small molecule tyrosine kinase inhibitor with dual inhibition of FMS-like receptor tyrosine kinase-3 (FLT3) and AXL. Gilteritinib is approved as monotherapy in patients with R/R *FLT3*-mutated AML based on results of the phase 3 ADMIRAL trial in which gilteritinib yielded superior overall survival (OS) compared to salvage chemotherapy (1). Gilteritinib is also being studied in the frontline setting in combination with hypomethylating agents with or without the addition of the B cell lymphoma 2 (BCL-2) inhibitor venetoclax, but gilteritinib is not currently approved as frontline therapy (2, 3). Ivosidenib is a small molecule inhibitor of Isocitrate Dehydrogenase 1 (IDH1). It is approved as monotherapy for treatment of R/R *IDH1*-mutated AML and as frontline therapy for patients with newly diagnosed *IDH1*-mutated AML who are ineligible for intensive chemotherapy based on early phase trials showing durable remissions in these patient populations (4, 5). Ivosidenib was also recently approved in combination with azacitidine as frontline therapy in patients with *IDH1*-mutated AML who are ineligible for intensive chemotherapy based on results of the phase III AGILE trial (6). Enasidenib is a small molecule inhibitor of Isocitrate Dehydrogenase 2 (IDH2) which is approved as monotherapy in patients with R/R *IDH2*-mutated AML based on studies demonstrating a favorable response rate and favorable OS in this setting (7, 8). The combination of frontline enasidenib + azacitidine has demonstrated a superior overall response rate compared to azacitidine alone in patients with *IDH2-*mutated AML ineligible for intensive chemotherapy, but enasidenib is not currently approved in the frontline setting (9). Lastly, the BCL2-inhibitor venetoclax can be utilized regardless of the AML mutation profile. Venetoclax is approved in combination with azacitidine or Low Dose Cytarabine (LDAC) as frontline therapy in patients who are ineligible for intensive chemotherapy based on results of the phase III VIALE-A and VIALE-C trials (10, 11).

Though all four of these agents have improved outcomes compared to previous standards of care, they are not considered curative therapies. Therefore, the need remains for allogeneic hematopoietic stem cell transplantation (allo-HSCT) as potentially curative therapy for European Leukemia Net (ELN) intermediate and high risk AML and for patients with R/R disease. The incidence of allo-HSCT has been reported in several, but not all, of the trials leading to the approval of these targeted agents and is variable ranging from 0-25% (1, 5, 7, 11). Most of these studies have not reported on post-transplant outcomes.

These novel targeted therapies are also being increasingly utilized in the frontline setting in patients of all ages and fitness levels, including patients who could otherwise be considered for intensive chemotherapy. This is due in part to the availability of these targeted therapies through ongoing clinical trials. These agents are also prescribed off-label in this setting, potentially due to provider and patient preference to help mitigate prolonged hospitalization and risk of regimen-related toxicities. The decision to use intensive chemotherapy vs. targeted therapy is particularly relevant in patients aged 60-75 due to the heterogeneity of this patient population with respect to disease characteristics, performance status, and comorbidities. However, the impact of targeted therapy vs. intensive chemotherapy on survival and response rates has not been extensively studied in this patient population. It is also unknown whether treatment with targeted vs. intensive therapy affects the ability to proceed to allo-HSCT based on achievement of a desirable response and impact on patient fitness. The impact of remission induction with targeted vs. intensive therapy on post-transplant outcomes has also not been studied in detail.

We conducted a single center, retrospective analysis to determine the impact of targeted therapy vs. intensive chemotherapy on outcomes in newly diagnosed patients with AML aged 60-75 including overall survival, treatment response, incidence of proceeding to allo-HSCT, and post-transplant outcomes. This study was initiated due to the expanding patient populations being treated with targeted therapies and the need to learn from real world experience.

## Materials and methods

### Patients and treatments

We performed a retrospective chart review for all newly diagnosed patients with AML treated at our center from 2016-2020 and included patients who were aged 60-75 at the time of diagnosis. Patients with Acute Promyelocytic Leukemia (APL), mixed phenotype leukemia, and acute leukemia of ambiguous lineage were excluded. The starting year was chosen as 2016 because this corresponds to the year in which recently approved targeted therapies started to become more widely utilized. Patients were only included in the study if they received either High Intensity Chemotherapy (HiC) or Lower Intensity Targeted Therapy (LITT) as their first prescribed AML treatment. HiC was defined as a regimen containing cytarabine and an anthracycline (daunorubicin or idarubicin) given on a 7 + 3-based schedule. LITT was defined as treatment with gilteritinib, ivosidenib, enasidenib, or venetoclax either as monotherapy or in combination with a hypomethylating agent (azacitidine or decitabine). Patients treated with a frontline hypomethylating agent alone were not included because this is becoming less common in clinical practice, and most patients treated with lower intensity therapies are now receiving targeted agents. All patients who received at least one dose of their first prescribed AML therapy were included in the analysis, as were patients who received more than one line of therapy for AML due to primary refractory disease, relapsed disease, or intolerance of initial treatment. Outcomes were compared between patients who received HiC any point during treatment (any HiC) and patients who did not (LITT only). For patients who underwent allo-HSCT, treatments received following transplant (e.g. maintenance therapy or treatment of relapse) did not count towards treatment group assignment. Salvage chemotherapy regimens containing any combination of an anthracycline, cytarabine, fludarabine, or etoposide such as MEC (mitoxantrone, etoposide, cytarabine) or FLAG (fludarabine, cytarabine, filgrastim) were considered as HiC for purposes of treatment group assignment. Patients who received other second or subsequent lines of treatment with therapies that did not qualify as HiC or LITT (e.g., hypomethylating agent monotherapy, other targeted therapies such as sorafenib, and other clinical trial therapies) were included in the analysis, but these other therapies did not count towards treatment group assignment.

### Definition of outcomes and statistical analyses

#### Patient characteristics

Baseline patient characteristics for the any HiC and LITT only groups were summarized using descriptive statistics. Wilcoxon test was used to compare continuous variables including age, Karnofsky Performance Status (KPS), baseline white blood cell (WBC) count, and baseline blast percentage. Chi-squared test was used to compare categorical variables including Eastern Cooperative Oncology Group (ECOG) performance status, baseline European Leukemia Net (ELN) risk classification, presence of therapy-related AML, presence of AML transformed from a prior myeloid neoplasm (transformed AML), and presence of *TP53*, *FLT* ITD, *FLT3* TKD, *IHD1* and *IDH2* mutations.

### Treatment response and overall survival

Response in this study was defined as complete remission (CR), complete remission with incomplete count recovery (CRi), or morphologic leukemia free state (MLFS). The definitions of these treatment responses are listed in **Supplementary Table 1**. The percentage of patients achieving at least one response with either initial or subsequent therapies was compared between the any HiC and LITT only groups. Response assessments for HiC therapies occurred either at count recovery or at approximately day 35, whichever occurred first. Response assessments for LITT therapies typically occurred after cycle 1 and cycle 2, then every 4-6 cycles thereafter or when there was concern for disease progression.

For the entire cohort, overall survival (OS) was calculated from the date of first AML therapy to the date of death from any cause, censoring patients who were alive at the time of last follow up. This primary OS endpoint was compared between the any HiC and LITT only groups. For the subgroup of patients who achieved at least one response during the course of therapy, a secondary OS was also calculated from the date of first response and compared between patients who received HiC vs. LITT as the treatment leading to the first response. The method of Kaplan-Meier was used to obtain estimates of OS, and the comparisons were conducted through log-rank test. Cox proportional hazards regression model was used to associate risk factors with the primary endpoint of OS from date of first AML therapy. Univariable models were first fit, then a backward selection procedure was followed to build a multivariable model. Variables were only left in the final model if they were significant at p ≤ 0.05 except for treatment intensity (any HiC vs. LITT only), which was forced in the model since this was the main variable of interest. Receipt of transplant was treated as a time-dependent variable in the Cox model.

### Incidence of transplant and post-transplant outcomes

The time to allo-HSCT was calculated from date of first AML therapy to the date of allo-HSCT, treating death without transplant as the competing risk. Time to acute and chronic graft vs. host disease (aGVHD and cGVHD) was defined from date of allo-HSCT to date of aGVHD or cGVHD onset, treating relapse or death without GVHD as the competing risk. The cumulative incidence rates (CIR) of allo-HSCT, aGVHD and cGVHD were estimated by cumulative incidence function and compared for patients receiving any HiC vs. LITT only using Gray’s test.

Beyond CIR of GVHD, other post-transplant outcomes included post-transplant OS and GVHD-and-relapse-free survival (GRFS). GRFS was defined as absence of grade 3-4 aGVHD, any chronic GVHD, relapse, and death from any cause. Post-transplant OS and GRFS were both calculated from date of transplant and analyzed by Kaplan-Meier method with log-rank test used to compare the any HiC and LITT only groups.

A separate comparison of post-transplant outcomes was also performed based on therapy received immediately prior to transplant (HiC vs. LITT).

## Results

### Patient characteristics

A total of 127 patients were included in the analysis. The any HiC group contained 73 patients. Sixty of the any HiC patients received HiC only. Twelve of the any HiC patients received HiC before LITT (5 achieved response with HiC then relapsed and received LITT, 3 were refractory to HiC then transitioned to LITT, and 4 achieved response with HiC then transitioned to LITT maintenance due to inability to tolerate additional chemotherapy). One of the any HiC patients received LITT before HiC (achieved response with LITT then relapsed and received HiC). The LITT only group contained 54 patients.

A comparison of baseline characteristics between the two treatment groups is shown in **Table 1**. Patients in the LITT only group were older (median age 68 vs. 66 years, p=0.0008), had a lower baseline white blood cell (WBC) count (median 5.6 vs. 13.1 K/µL, p=0.006), a trend toward more adverse ELN risk classification (19.6% favorable, 21.6% intermediate, and 58.8% adverse vs. 34.9% favorable, 21.2% intermediate, and 43.9% adverse, p=0.16), a higher frequency of therapy related AML (14.8% vs. 5.5%, p=0.08), and a higher frequency of transformed AML (25.9% vs. 9.6%, p=0.01). The LITT only group also had a higher frequency of *TP53* mutations (18.9% vs. 7.1%, p=0.05), a lower frequency of *FLT3* ITD mutations (9.4% vs. 31%, p=0.004), and a higher frequency of *IDH2* mutations (46.3% vs. 14.3%, p<0.001). Baseline performance status notably did not differ significantly between the two groups.

**Table 1:** Baseline patient characteristics according to treatment group.

%	Overall (N=127)	LITT only (N=54)	Any HiC (N=73)	p-value
Age at Treatment Start, median (range)	66 (61-75)	68 (61-75)	66 (61-75)	0.0008
Sex				0.88
Female	40.0	40.7	39.4	
Male	60.0	59.3	60.6	
KPS, median (range)	80 (30-100)	80 (30-100)	80 (30-100)	0.46
ECOG PS				0.57
0	27.1	26.4	27.5	
1	59.0	54.7	62.3	
2	12.3	17.0	8.7	
3	1.6	1.9	1.5	
PB WBC at Diagnosis x 10^3^/µL, median (range)	7.9 (0.6-426)	5.6 (0.8-178.3)	13.1 (0.6-426)	0.006
PB Blast% at Diagnosis, median (range)	21.5 (0-97)	27 (0-95)	20 (0-97)	0.68
ELN Risk				0.16
Favorable	28.2	19.6	34.9	
Intermediate	21.4	21.6	21.2	
Adverse	50.4	58.8	43.9	
Therapy related AML	9.5	14.8	5.5	0.08
Transformed AML	16.5	25.9	9.6	0.01
*TP53* mutated	12.2	18.9	7.1	0.05
*FLT3* ITD mutated	21.8	9.4	31.0	0.004
*FLT3* TKD mutated	8.8	11.1	7.0	0.53
*IDH1* mutated	9.6	9.3	9.9	0.91
*IDH2* mutated	28.2	46.3	14.3	<.0001

LITT, Low Intensity Targeted Therapy; HiC, High Intensity Chemotherapy; KPS, Karnofsky Performance Status; ECOG PS, Eastern Cooperative Oncology Group Performance Status; PB, Peripheral Blood; WBC, White Blood Cell Count; ELN, European Leukemia Net; FLT3, FMS-Like Tyrosine Kinase 3; ITD, Internal Tandem Duplication; TKD, Tyrosine Kinase Domain; IDH1, Isocitrate Dehydrogenase 1; IDH2, Isocitrate Dehydrogenase 2.

### Treatment response and overall survival

**Figure 1:**
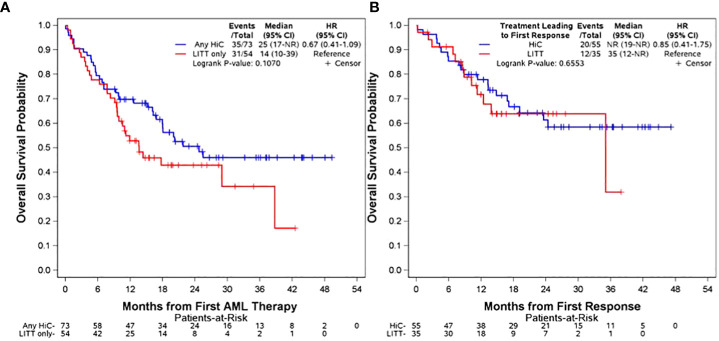
Overall Survival Panel **(A)**: OS from date of treatment initiation stratified by any HiC vs. LITT only. Panel **(B)** OS from date of first response stratified by treatment leading to first response (HiC vs. LITT). The distributions were estimated for each treatment group with the use of the Kaplan-Meier method and were compared with the log-rank test. The hazard ratio for death was estimated with the use of the Cox proportional-hazards model.

Clinical outcomes are presented in **Table 2**. A total of 60 patients (82%) in the any HiC group achieved at least one response compared to 33 patients (61%) in the LITT only group (p=0.008). The analysis of primary OS from date of first AML therapy is depicted in row 2 of **Table 2** and in panel A of **Figure 1**. There was no significant difference in median OS between the any HiC and LITT only groups. With a median follow-up of 21.6 months (range: 9.1-49.3) among survivors in the entire cohort, median OS in the any HiC group was 24.7 months (95% CI 16.8-NR) compared to 13.6 months (95% CI 9.6-38.7) in the LITT only group (hazard ratio (HR) for death, 0.67; 95% CI, 0.41 to 1.09; p=0.11). Early mortality also did not differ significantly between the any HiC and LITT only groups. Mortality within 30 days of treatment initiation was 4.1% in the any HiC group vs. 5.6% in the LITT only group (p=0.70). Mortality within 60 days of treatment initiation was 9.6% in the any HiC group vs. 9.3% in the LITT only group (p=1.00). Panel B of **Figure 1** depicts the Kaplan Meier curves for OS from the date of first response for patients achieving their first response with HiC vs. LITT. There was no significant difference in post-response OS between these two groups.

**Table 2:** Clinical outcomes according to treatment group.

	Overall (N=127)	LITT only (N=54)	Any HiC (N=73)	p-value
Achieved Response, N (%)	93 (73.2)	33 (61.1)	60 (82.2)	0.008
OS from first AML therapy				0.11
Number of deaths	66	31	35	
Median in months (95% CI)	20.0 (14.4-38.7)	13.6 (9.6-38.7)	24.7 (16.8-NR)	
Median follow-up in months (range)	21.6 (9.1-49.3)	19.0 (9.5-42.5)	27.3 (9.1-49.3)	
Allo-HSCT				0.0003
Number of transplants	53	13	40	
6-month CIR (95% CI)	29.1% (21.5-37.2%)	11.1% (4.5-21.2%)	42.5% (30.9-53.5%)	
12-month CIR (95% CI)	37.9% (29.4-46.3%)	20.4% (10.8-32.1%)	50.8% (38.7-61.8%)	
OS from allo-HSCT	**(N=53)**	**(N=13)**	**(N=40)**	0.71
Number of deaths	16	3	13	
6-month estimate (95% CI)	86.2% (73.1-93.2%)	92.3% (56.6-98.9%)	84.4% (68.4-92.7%)	
12-month estimate (95% CI)	74.6% (59.4-84.8%)	82.1% (44.4-95.3%)	72.3% (54.5-84.1%)	
aGVHD				0.41
Number of events	33	10	23	
1-month CIR (95% CI)	26.4% (15.4-38.8%)	23.1% (5.1-48.5%)	27.5% (14.7-41.9%)	
3-month CIR (95% CI)	56.6% (42.1-68.8%)	61.5% (28.6-82.9%)	55.0% (38.1-69.0%)	
cGVHD				0.31
Number of events	21	6	15	
6-month CIR (95% CI)	18.5% (9.0-30.6%)	18.5% (2.5-46.2%)	18.4% (8.0-32.2%)	
12-month CIR (95% CI)	44.5% (29.4-58.5%)	60.7% (21.1-85.2%)	39.6% (23.3-55.6%)	
GRFS				0.36
Number of events	35	9	26	
Median in months (95% CI)	6.2 (4.7-10.3)	5.6 (4.1-10.9)	6.5 (4.4-12.0)	

OS, Overall Survival; CIR, Cumulative Incidence Rate; Allo-HSCT, allogeneic hematopoietic stem cell transplantation; aGVHD, Acute GVHD; cGVHD, Chronic GVHD; GRFS, GVHD and Relapse Free Survival, defined as grade 3-4 aGVHD-free, cGVHD-free and relapse-free survival.

Cox regression analysis of OS from date of first AML therapy is depicted in **Table 3**. The left side of the table shows estimates from the univariable models. Higher ELN risk classification and the presence of *TP53* mutations were found to be associated with inferior OS, and receipt of allo-HSCT was highly predictive of superior OS on univariable analyses. Treatment intensity, chronological age, baseline performance status, therapy-related AML, and transformed AML were not found to be correlated with OS in this cohort of patients. The right side of the table presents the multivariable model. ELN risk classification, *TP53* mutation status, and receipt of transplant all remained significantly associated with OS after adjusting for treatment intensity and other variables.

**Table 3:** Cox regression analysis of os from first aml therapy (N=127).

66 events	Univariable models		Multivariable model	
	Hazard ratio (95% CI)	p-value	Hazard ratio (95% CI)	p-value
Any HiC vs. LITT only	0.67 (0.41-1.09)	0.11	1.50 (0.84-2.68)	0.17
Age at Treatment Start,5 year increase	1.00 (0.74-1.34)	0.98	‐‐‐‐	‐‐‐‐
Male vs. Female	1.01 (0.62-1.67)	0.96	‐‐‐‐	‐‐‐‐
KPS, 10-unit increase	1.00 (0.82-1.21)	0.97	‐‐‐‐	‐‐‐‐
ECOG, 1-unit increase	1.05 (0.72-1.52)	0.82	‐‐‐‐	‐‐‐‐
WBC at Diagnosis, 10-unit increase	1.00 (0.97-1.04)	0.94	‐‐‐‐	‐‐‐‐
Blast Percentage, 10% increase	1.00 (0.92-1.08)	0.97	‐‐‐‐	‐‐‐‐
ELN Risk (vs. Favorable)				
Intermediate	1.64 (0.71-3.79)	0.25	1.87 (0.79-4.43)	0.16
Adverse	3.36 (1.67-6.74)	0.0007	3.18 (1.49-6.79)	0.0027
Therapy related AML vs. Not	1.82 (0.86-3.83)	0.12	‐‐‐‐	‐‐‐‐
Secondary AML vs. Not	1.06 (0.55-2.02)	0.87	‐‐‐‐	‐‐‐‐
*TP53* mutated vs. unmutated	3.18 (1.72-5.89)	0.0002	2.42 (1.20-4.90)	0.01
*FLT3* ITD mutated vs. unmutated	0.91 (0.48-1.70)	0.76	‐‐‐‐	‐‐‐‐
*FLT3* TKD mutated vs. unmutated	1.36 (0.62-2.99)	0.44		
*IDH1* mutated vs. unmutated	0.43 (0.16-1.20)	0.11	‐‐‐‐	‐‐‐‐
*IDH2* mutated vs. unmutated	1.14 (0.67-1.96)	0.63	‐‐‐‐	‐‐‐‐
Transplant^#^	0.20 (0.11-0.37)	<.0001	0.17 (0.08-0.34)	<.0001

^#^Entered model as a time-dependent variable.

‐‐‐‐Indicates lack of statistical significance in multivariable analysis.

HiC, High Intensity Chemotherapy; LITT, Low Intensity Targeted Therapy; KPS, Karnofsky Performance Status; ECOG, Eastern Cooperative Oncology Group; WBC, White Blood Cell; ELN, European Leukemia Net; FLT3, FMS-Like Tyrosine Kinase 3; ITD, Internal Tandem Duplication; TKD, Tyrosine Kinase Domain; IDH1, Isocitrate Dehydrogenase 1; IDH2, Isocitrate Dehydrogenase 2.

### Incidence of transplant and post-transplant outcomes

The CIR of allo-HSCT and post-transplant outcomes for the any HiC and LITT only groups are also presented in **Table 2**. The incidence of allo-HSCT was significantly higher in the any HiC group compared to the LITT only group (6-month CIR of 42.5% vs. 11.1%, p=0.0003). However, post-transplant OS, incidence of aGVHD, incidence of cGVHD, and GRFS did not differ significantly between the any HiC and LITT only groups.

Of the 53 patients who underwent allo-HSCT, 45 received either HiC or LITT as the treatment immediately prior to transplant. Immediate pre-transplant therapy in the remaining eight patients consisted of decitabine monotherapy in six patients, intensive chemotherapy plus a clinical trial agent in one patient, and sorafenib plus azacitidine in one patient. **Supplementary Table 2** shows a comparison of post-transplant outcomes for patients whose immediate pre-transplant therapy was HiC vs. LITT. None of the post-transplant outcomes included in our analysis differed significantly between these two groups.

## Discussion

The findings from this study demonstrate that baseline genomic features and receipt of transplant were the most important predictors of OS in this cohort of patients with AML aged 60-75. These were the only variables that were independently predictive of survival, and they were notably more important than treatment intensity and chronological age. Median OS from initiation of AML therapy was 13.6 months (95% CI 9.6-38.7) in the LITT only group in this study. This compares to median OS of 8.8-24 months in previously published clinical trials involving these targeted therapies (1, 2, 4, 6, 8, 10, 12). Median OS from initiation of AML therapy did not differ significantly between the any HiC and LITT only groups in this study. Other retrospective studies comparing intensive chemotherapy to venetoclax + hypomethylating agent therapy have also found that intensive chemotherapy was not associated with superior OS. One study in patients over age 60 comparing frontline venetoclax + decitabine to intensive chemotherapy found that the venetoclax + decitabine group had superior median OS (12.4 vs. 4.5 months, HR = 0.48, 95%CI 0.29-0.79, *P* < .01) using a propensity-matched comparison (13). Another study found that frontline venetoclax + azacitidine demonstrated a trend toward superior OS compared to frontline intensive chemotherapy in patients of all ages using propensity-matched cohorts (14).

In regards to treatment response, 61% of the patients in the LITT only group achieved at least one response during the course of therapy. This compares to response rates of 30.4%-74% in clinical trials involving these targeted therapies (1, 2, 4–7, 9, 10, 12). It was noted that a higher percentage of patients in the any HiC group achieved at least one response compared to patients in the LITT only group in our study. This is likely due in part to the more adverse baseline cytogenetic and molecular features in the LITT only group.

The incidence of transplant was higher amongst patients who received intensive chemotherapy in our study, but post-transplant outcomes did not differ significantly based on treatment intensity utilized for response induction. Post-response overall survival was also similar in patients who achieved their first response with intensive chemotherapy vs. targeted therapy. These findings suggest that patients who follow the standard treatment paradigm of response induction followed by transplant have similar survival outcomes regardless of whether response is achieved with intensive vs. targeted therapy. A follow up analysis from the ADMIRAL trial similarly found comparable median post-transplant OS for patients who had been treated with salvage gilteritinib vs. standard of care chemotherapy (16.1 months for gilteritinib vs. 15.3 months for standard of care chemotherapy; HR=1.076; 95% CI: 0.536-2.160) (15). The 12 month post-transplant OS was 82% for the LITT only group in our study. Other retrospective analyses have similarly demonstrated favorable post-transplant OS in patients treated with venetoclax + hypomethylating agents with 12 month post-transplant OS of 68-78% (16, 17). Receipt of transplant was highly protective in our study and associated with improved OS. This highlights the vital role of transplant in this population of older patients with AML regardless of the treatment intensity utilized to induce response. A recently published CIBMTR analysis examining predictors of post-transplant outcomes in patients with AML aged 60 and older similarly found that age alone was not a barrier to transplant success and should not exclude patients from undergoing allo-HSCT (18).

Another important question regarding treatment response that was not addressed in this study is the impact of targeted vs. intensive chemotherapy on achievement of measurable residual disease (MRD) negativity. The achievement of MRD negativity by multiparametric flow cytometry (MFC), polymerase chain reaction (PCR), and Next Generation Sequencing (NGS) has been shown to be associated with improved OS and disease free survival in patients with AML treated with intensive chemotherapy (19). There is increasing evidence that MRD negativity has prognostic significance in patients treated with targeted therapies as well (20, 21). Pre-transplant MRD negativity by MFC, PCR, or NGS has also been shown to be predictive of superior post-transplant outcomes including improved OS, improved leukemia free survival (LFS), and reduced incidence of relapse in patients of all ages (22, 23). However, findings from recent studies have also suggested that pre-transplant MRD status by flow or NGS may not be independently predictive of post-transplant LFS or OS in patients older than 60, particularly in patients who are treated with lower intensity therapy for remission induction (24, 25). The impact of treatment intensity on achievement of MRD negativity in this patient population will be an important question for future studies to address, as will the impact of MRD negativity on post-transplant outcomes for patients treated with intensive vs. targeted therapies. Addressing these questions in a retrospective study is challenging due to lack of standardization in terms of time points and methods for MRD assessments among the patients included. Prospective studies in which this can be standardized would be needed to effectively address this. In our study, it is notable that post-response OS did not differ significantly in patients achieving their first response with HiC vs. LITT regardless of response depth.

There are other limitations to consider in this study as well. The retrospective, single center design is one limiting factor. Provider and patient bias represent a limitation given that the factors determining whether patients received HiC vs. LITT were not standardized in this retrospective study. Many of the patients in the LITT only group had been treated on clinical trials and therefore may not be fully representative of patients treated with targeted therapies in the real world in terms of performance status or comorbidities. Another limitation is the relatively small sample size which is particularly notable for the subset of patients who underwent transplant when considering the analysis of post-transplant outcomes. Patients were also not screened for transplant candidacy which limits the ability to draw conclusions regarding the impact of treatment intensity on ability to proceed to transplant. The heterogeneity that exists in this study with regards to patient characteristics, disease characteristics, and treatments received represents another limitation.

Ultimately, decisions regarding optimal treatment intensity in this patient population remain challenging due to the heterogeneity that exists regarding patient and disease characteristics. Validated tools exist which can help to guide this decision. The AML-Composite Model (AML-CM) is one such tool that combines patient comorbidities, age, and cytogenetic/molecular risk to predict one year mortality (26). Another recently published study found that patients treated with intensive chemotherapy can be divided into different groups with distinct 4-week mortality rates based on age, organ dysfunction, frailty, cytogenetic abnormalities, and the presence of infection (27). Providers often prefer to use lower intensity therapies in this age group whenever possible in order to avoid toxicity and prolonged hospitalizations, even in patients who may otherwise be eligible for intensive chemotherapy. Robust comparisons of intensive vs. targeted therapies are therefore necessary to determine whether intensive chemotherapy can be deferred in favor of targeted therapies without compromising patient outcomes. The gold standard for addressing this question would be to conduct prospective, randomized trials comparing intensive chemotherapy to targeted therapy in patients who are considered to be eligible for intensive chemotherapy and potential candidates for transplant at baseline. These types of studies should focus on specific molecular subsets of AML in order to limit heterogeneity and provide better understanding of whether certain molecular subsets are more likely to benefit from intensive vs. targeted therapies. It may be difficult to adequately recruit participants for this type of study due to patient and physician bias regarding preferred treatment intensity, but the results would be helpful for informing clinical practice. There are currently ongoing prospective trials comparing intensive chemotherapy to venetoclax + hypomethylating agents in adults aged 18 to 59 (NCT05177731) and adults aged 18 and older (NCT05177731) who are considered eligible for intensive chemotherapy. The results of these studies will be informative, but the creation of similar trials specifically for patients aged 60 and older would help to further guide treatment decisions for this patient subset. The question of optimal treatment intensity is also likely to evolve over time as more targeted therapies are introduced and as they become increasingly utilized in different combinations.

## Data availability statement

The raw data supporting the conclusions of this article will be made available by the authors, without undue reservation.

## Ethics statement

The studies involving human participants were reviewed and approved by Cancer Institutional Review Board of The Ohio State University. Written informed consent for participation was not required for this study in accordance with the national legislation and the institutional requirements.

## Author contributions

KS contributed to data gathering, data analysis, manuscript writing, and manuscript editing. YH contributed to statistical design, statistical analysis, and manuscript editing. MR contributed to data analysis and manuscript writing. PE contributed to data gathering and manuscript review. AM contributed to manuscript review and manuscript editing. SW served as senior author and contributed to data analysis, manuscript review, and manuscript editing.

## Conflict of interest

AM reports the following disclosures: Scientific Advisory Board: Astellas, AbbVie, Genentech, Syndax Pharmaceuticals, Servier, Zentalis, and Ryvu. DSMC Involvement: Jazz Pharmaceuticals and Daiichi Saynko.

The remaining authors declare that the research was conducted in the absence of any commercial or financial relationships that could be construed as a potential conflict of interest.

## Publisher’s note

All claims expressed in this article are solely those of the authors and do not necessarily represent those of their affiliated organizations, or those of the publisher, the editors and the reviewers. Any product that may be evaluated in this article, or claim that may be made by its manufacturer, is not guaranteed or endorsed by the publisher.

## References

[1] Perl AE Martinelli G Cortes JE Neubauer A Berman E Paolini S. Gilteritinib or chemotherapy for relapsed or refractory FLT3-mutated AML. New Engl J Med (2019) 381(18):1728–40. doi: 10.1056/NEJMoa1902688 31665578

[2] WangESMontesinosPMindenMDLeeJHeuserMNaoeT. Phase 3 trial of gilteritinib plus azacitidine vs azacitidine for newly diagnosed FLT3mut+ AML ineligible for intensive chemotherapy. Blood (2022) 39(24):3546–57. doi: 10.1182/blood.2021014586 35917453

[3] Maiti A DiNardo CD Daver NG Rausch CR Ravandi F Kadia TM. Triplet therapy with venetoclax, FLT3 inhibitor and decitabine for FLT3-mutated acute myeloid leukemia. Blood Cancer J (2021) 11(2):25. doi: 10.1038/s41408-021-00410-w 33563904 PMC7873265

[4] DiNardo CD Stein EM Botton S Roboz GJ Altman JK Mims AS. Durable remissions with ivosidenib in IDH1-mutated relapsed or refractory AML. New Engl J Med (2018) 378(25):2386–98. doi: 10.1056/NEJMoa1716984 29860938

[5] Roboz GJ Dinardo CD Stein EM Botton S Mims AS Prince GT. Ivosidenib (IVO; AG-120) in IDH1-mutant newly-diagnosed acute myeloid leukemia (ND AML): Updated results from a phase 1 study. J Clin Oncol (2019) 37(15_suppl):7028. doi: 10.1200/JCO.2019.37.15_suppl.7028

[6] Montesinos P Recher C Vives S Zarzycka E Wang J Bertani G. Ivosidenib and azacitidine in IDH1-mutated acute myeloid leukemia. New Engl J Med (2022) 386(16):1519–31. doi: 10.1056/NEJMoa2117344 35443108

[7] Stein EM DiNardo CD Pollyea DA Fathi AT Roboz GJ Altman JK. Enasidenib in mutant IDH2 relapsed or refractory acute myeloid leukemia. Blood (2017) 130(6):722–31. doi: 10.1182/blood-2017-04-779405 PMC5572791 28588020

[8] de Botton S Brandwein JM Wei AH Pigneux A Quesnel B Thomas X. Improved survival with enasidenib versus standard of care in relapsed/refractory acute myeloid leukemia associated with IDH2 mutations using historical data and propensity score matching analysis. Cancer Med (2021) 10(18):6336–43. doi: 10.1002/cam4.4182 PMC8446562 34427990

[9] DiNardo CD Schuh AC Stein EM Montesinos P Wei AH Botton S. Enasidenib plus azacitidine versus azacitidine alone in patients with newly diagnosed, mutant-IDH2 acute myeloid leukaemia (AG221-AML-005): A single-arm, phase 1b and randomised, phase 2 trial. Lancet Oncol (2021) 22(11):1597–608. doi: 10.1016/S1470-2045(21)00494-0 34672961

[10] DiNardo CD Jonas BA Pullarkat V Thirman MJ Garcia JS Wei AH. Azacitidine and venetoclax in previously untreated acute myeloid leukemia. New Engl J Med (2020) 383(7):617–29. doi: 10.1056/NEJMoa2012971 32786187

[11] Wei AH Montesinos P Ivanov V DiNardo CD Novak J Laribi K. Venetoclax plus LDAC for newly diagnosed AML ineligible for intensive chemotherapy: A phase 3 randomized placebo-controlled trial. Blood (2020) 135(24):2137–45. doi: 10.1182/blood.2020004856 PMC7290090 32219442

[12] Wang ES Montesinos P. Phase 3, open-label, randomized study of gilteritinib and azacitidine vs azacitidine for newly diagnosed FLT3-mutated acute myeloid leukemia in patients ineligible for intensive induction chemotherapy. Blood (2021) 138:700. doi: 10.1182/blood-2021-145379

[13] Maiti A Qiao W Sasaki K Ravandi F Kadia TM Jabbour EJ. Venetoclax with decitabine vs intensive chemotherapy in acute myeloid leukemia: A propensity score matched analysis stratified by risk of treatment-related mortality. Am J Hematol (2021) 96(3):282–91. doi: 10.1002/ajh.26061 PMC8128145 33264443

[14] Cherry EM Abbott D Amaya M McMahon C Schwartz M Rosser J. Venetoclax and azacitidine compared with induction chemotherapy for newly diagnosed patients with acute myeloid leukemia. Blood Adv (2021) 5(24):5565–73. doi: 10.1182/bloodadvances.2021005538 PMC8714726 34610123

[15] Perl AE Larson RA Podoltsev NA Strickland S Wang ES Schiller GJ. Follow-up of patients with FLT3-mutated R/R AML in the phase 3 ADMIRAL trial. J Clin Oncol (2021) 39(15_suppl):7013. doi: 10.1200/JCO.2021.39.15_suppl.7013

[16] Pratz KW DiNardo CD Arellano ML Letai AG Thirman M Pullarkat VA. Outcomes after stem cell transplant in older patients with acute myeloid leukemia treated with venetoclax-based therapies. Blood (2019) 134(Supplement_1):264. doi: 10.1182/blood-2019-127251 41510611

[17] Kennedy VE Hui G Gaut D Mittal V Oliai C Muffly LS. Hypomethylating agents in combination with venetoclax as a bridge to allogeneic transplant in acute myeloid leukemia. Blood (2020) 136(Supplement 1):32–3. doi: 10.1182/blood-2020-143002

[18] Maakaron JE Zhang M-J Chen K Abhyankar S Bhatt VR Chhabra S. Age is no barrier for adults undergoing HCT for AML in CR1: Contemporary CIBMTR analysis. Bone Marrow Transplant (2022) 57(6):911–7. doi: 10.1038/s41409-022-01650-5 PMC9232949 35368040

[19] Short NJ Zhou S Fu C Berry DA Walter RB Freeman SD. Association of measurable residual disease with survival outcomes in patients with acute myeloid leukemia: A systematic review and meta-analysis. JAMA Oncol (2020) 6(12):1890–9. doi: 10.1001/jamaoncol.2020.4600 PMC7545346 33030517

[20] Pratz KW Jonas BA Pullarkat V Recher C Schuh AC Thirman MJ. Measurable residual disease response and prognosis in treatment-naïve acute myeloid leukemia with venetoclax and azacitidine. J Clin Oncol (2022) 40(8):855–65. doi: 10.1200/JCO.21.01546 PMC8906463 34910556

[21] Altman JK Perl AE Cortes JE Smith CC Litzow MR Hill JE. Deep molecular response to gilteritinib to improve survival in FLT3 mutation-positive relapsed/refractory acute myeloid leukemia. J Clin Oncol (2017) 35(15_suppl):7003. doi: 10.1200/JCO.2017.35.15_suppl.7003

[22] Buckley SA Wood BL Othus M Hourigan CS Ustun C Linden MA. Minimal residual disease prior to allogeneic hematopoietic cell transplantation in acute myeloid leukemia: A meta-analysis. Haematologica (2017) 102(5):865–73. doi: 10.3324/haematol.2016.159343 PMC5477605 28126965

[23] Press RD Eickelberg G Froman A Yang F Stentz A Flatley EM. Next-generation sequencing-defined minimal residual disease before stem cell transplantation predicts acute myeloid leukemia relapse. Am J Hematol (2019) 94(8):902–12. doi: 10.1002/ajh.25514 31124175

[24] Murdock HM Kim HT Denlinger N Vachhani P Hambley B Manning BS. Impact of diagnostic genetics on remission MRD and transplantation outcomes in older AML patients. Blood (2022) 39(24):3546–57. doi: 10.1182/blood.2021014520 PMC9203701 35286378

[25] Hilberink JR Morsink LM Velden der WJFM Mulder AB Hazenberg CLE Groot M. Pretransplantation MRD in older patients with AML after treatment with decitabine or conventional chemotherapy. Transplant Cell Ther (2021) 27(3):246–52. doi: 10.1016/j.jtct.2020.12.014 33781523

[26] Sorror ML Storer BE Fathi AT Gerds AT Medeiros BC Shami P. Development and validation of a novel acute myeloid leukemia-composite model to estimate risks of mortality. JAMA Oncol (2017) 3(12):1675–82. doi: 10.1001/jamaoncol.2017.2714 PMC5824273 28880971

[27] Sasaki K Kadia T Begna K DiNardo CD Borthakur G Short NJ. Prediction of early (4-week) mortality in acute myeloid leukemia with intensive chemotherapy. Am J Hematol (2022) 97(1):68–78. doi: 10.1002/ajh.26395 34716921 PMC11809073

