# Supplementary material for: Survival, response rates, and post-transplant outcomes in patients with Acute Myeloid Leukemia aged 60-75 treated with high intensity chemotherapy vs. lower intensity targeted therapy
Source: Front Oncol. 2022 Oct 3;12:1017194. doi: 10.3389/fonc.2022.1017194 (PMC9574198; doi:10.3389/fonc.2022.1017194)
Supplement: Supplementary file 1 [file Table_1.docx]

Supplementary Material

**Supplementary Table 1: Definitions of Treatment Responses**

Abbreviations: CR: Complete Response; CRi: Complete Response with Incomplete Count Recovery; MLFS: Morphologic Leukemia Free State; ANC: Absolute Neutrophil Count; K: Thousand; plts: platelets

| **Response** | **Definition** |
| --- | --- |
| CR | <5% medullary blasts detected by cytomorphologic assessment, absence of circulating blasts, absence of extramedullary disease, ANC≥1K/µL, and plts≥100K/µL |
| CRi | Same as CR except with either ANC<1K/µL OR plts<100K/µL |
| MLFS | Same as CR except with ANC<1K/µL AND plts<100K/µL |

**Supplementary Table 2: Post-Transplant Outcomes Based on Immediate Pre-Transplant Therapy**

Abbreviations: OS: Overall Survival; CIR: Cumulative Incidence Rate; Allo-HSCT: allogeneic hematopoietic stem cell transplantation; aGVHD: Acute GVHD; cGVHD: Chronic GVHD; GRFS: GVHD and Relapse Free Survival, defined as grade 3-4 aGVHD-free, cGVHD-free and relapse-free survival

|  | LITT  (N=15) | HiC  (N=30) | p-value |
| --- | --- | --- | --- |
| OS from time of allo-HSCT  Number of deaths  6-month estimate (95% CI)  12-month estimate (95% CI) | 3  93.3% (61.3-99.0)  83.0% (45.7-95.6) | 6  89.9% (71.8-96.6)  82.3% (62.6-92.3) | 0.67 |
| aGVHD  Number of events  1-month CIR (95% CI)  3-month CIR (95% CI) | 12  26.7% (7.7-50.5)  66.7% (35.3-85.4) | 15  23.3% (10.1-39.7)  46.7% (28.0-63.4) | 0.10 |
| cGVHD  Number of events  6-month CIR (95% CI)  12-month CIR (95% CI) | 6  16.5% (2.3-42.5)  56.3% (20.0-81.5) | 13  17.3% (6.1-33.2)  43.7% (24.5-61.5) | 0.57 |
| GRFS  Number of events  Median in months (95% CI) | 10  5.6 (2.8-10.9) | 18  9.1 (5.0-NR) | 0.11 |
